# Structural Analysis of Humic Acid in Soil at Different Corn Straw Returning Modes through Fluorescence Spectroscopy and Infrared Spectroscopy

**DOI:** 10.1155/2019/1086324

**Published:** 2019-12-29

**Authors:** JinFeng Gao, Sen Dou, ZhiGuo Wang

**Affiliations:** ^1^College of Resource and Environment, Jilin Agricultural University, Changchun 130118, China; ^2^Beihua University, Jilin City 132013, China; ^3^Jilin City Academy of Agricultural Sciences, Jilin City 132000, China

## Abstract

The purpose of this study is to analyze the effects of different straw returning modes on the structure of humic acid (HA) in soil by fluorescence spectroscopy and infrared (IR) spectroscopy. Four different straw returning modes, including straw returning to topsoil (St), straw returning to subsoil (Ss), straw mixing with topsoil (Smt), and straw mixing with subsoil (Sms), were used in this study; the soil HA was analyzed after 12 months of corn straw returning by a combination of fluorescence spectroscopy and IR spectroscopy. Based on the results, it was established that IR spectroscopy can estimate the complication and oxidation degree of soil HA and also evaluate its aliphaticity and aromaticity. Monodimensional fluorescence spectroscopy could preliminarily determine the changes in the humification of HA through the fluorescence intensities. The intensity ratio of I_456_/I_380_ calculated from synchronous-scan fluorescence spectra could be used to evaluate the humification degree of soil HA. The total luminescence spectra of HAs provided more information on the fluorophores in the structure, including the amount and peak position of lignin-like structures and phenol-like or naphthol-like structures. Among the four straw returning modes, Ss is the most beneficial for reducing the oxidation degree and increasing the aromatization and humification degree of subsoil HA. It is believed that fluorescence spectroscopy and IR spectroscopy are relatively simple and sensitive methods for analyzing soil HA.

## 1. Introduction

Crop straw is a good source of soil organic fertilizers [1–4]. At present, straw returning is the most convenient and practical straw utilization technology [5, 6]. The effect of straw returning on soil organic matter (SOM) is not only reflected in the change in organic carbon content but also, more importantly, can improve the quality of SOM [7]. Scholars [8] usually divided the 0–40 cm soil layer into topsoil (0–20 cm) and subsoil (20–40 cm). Most of the straw returned to the field is applied to the topsoil, and extensive research on the topsoil has been conducted. Tian et al. [9] buried wheat straw in the topsoil, and the organic carbon content in the topsoil increased significantly. Thomsen and Christensen [10] studied the long-term accumulation of wheat straw in the topsoil and reported that the content of SOM increased by 15.4%. Bhattacharyya et al. [11] studied that rice straw mixing with topsoil can markedly increase the total carbon content of the soil. Lenka and Lal [12] carried out a straw-covered no-tillage rotation for 15 consecutive years, during which the organic matter content of the 0–10 cm soil layer increased by about 50%. Furthermore, there are some other studies on straw returning, which were mostly concentrated on the soil organic carbon (SOC) content of the topsoil [13, 14]. In fact, more than 50% of SOC content is stored in the 30–100 cm soil layer [15]; the fertility of the subsoil also has a certain impact on crop yield [16]. The above scholars have studied SOM-related properties after straw returning through various modes. These studies mainly focus on topsoil straw returning.

Soil humic substance (HS) is the main component of SOM, accounting for 85–90% of the total organic matter of the soil, including humic acid (HA), fulvic acid (FA), and humin (HM) [17]. HA is an active substance in HS and an important indicator of soil fertility. The changes in the composition, structure, and properties of HA are directly related to soil fertilization properties. Straw returning can improve soil fertility and increase SOM, and different modes of straw returning have a great influence on the structure of soil HA. Wu et al. [18] found that at the later stage of corn straw composting, the lower organic matters such as phenol, quinones, soluble organic carbon, and more complex compound had incorporated in the aromatic structure of HS, leading to the increase of HA concentration. Furthermore, most studies focus on exploring the changes in the properties of SOM after straw returning, but there are few studies on the structural characteristics of soil HA. Therefore, further study on the structural changes of soil HA after straw returning is important for the regeneration of SOM and the research of soil fertility.

At the molecular level, HA has a large number of unsaturated fatty acid chains and aromatic structures with various functional groups. The research of Wu et al. [19] shows that precursors (including polyphenols, carboxyl, amino acids, and polysaccharide) are involved in the formation of HA. Therefore, these structures can be analyzed through fluorescence spectroscopy. Fluorescence spectroscopy has the advantages of high sensitivity, strong selectivity, and no damage to the sample; thus, researchers have gradually inclined toward using it to analyze the structure, configuration, and kinetics of HA interactions with molecules and intramolecular interactions [20–22]. Researchers have demonstrated the potential of fluorescence spectroscopy for the evaluation of the humification and molecular size of humic substances [23]. Milori et al. [24] used monodimensional fluorescence spectroscopy to evaluate the humification degree of 18 HAs extracted from four Brazilian soils under different land use, tillage, or cropping systems. He chose 465 nm as the excitation source to obtain the emission spectra and determined the degree of humification of 18 HAs through the intensity ratio of 465 nm and 400 nm calculated from the synchronous-scan spectra at Δ*λ* = 55 nm. In the fluorescence spectroscopic analysis of the topsoil and subsoil HA of a clayey Brazilian oxisol soil, Bertoncini et al. [25] found a peak at 546 nm in its emission spectra and a peak at 457 nm in its excitation spectra. The emission wavelength of the topsoil HA was red-shifted compared with that of the subsoil. Total luminescence (TL) fluorescence spectroscopy, in the form of excitation and emission matrix (EEM), is performed by changing both the excitation and emission wavelengths and providing intensity information. Compared with monodimensional fluorescence spectroscopy (emission, excitation, and synchronous-scan), EEM fluorescence spectroscopy is more effective in distinguishing compounds with different fluorescent properties in HA [26, 27]. Enev et al. [28] obtained the EEM fluorescence spectra of HA isolated from five Czech soil types and found that the fluorescence peaks of different types of soil HA are similar but the peak intensity is different. Antízar–Ladislao et al. [29] investigated the HA of an aged coal-tar contaminated soil using EEM fluorescence spectroscopy; this method provided evidence of progressive humification of the composting mixture through peak wavelength shifts and fluorescence intensity. Fasurová and Pospíšilová [30] studied three types of soil HA using EEM fluorescence spectroscopy and observed three peaks. They considered that there should be at least two or more fluorescent substances in the soil HA. Santos et al. [31] used EEM fluorescence spectroscopy to analyze the structure of HAs from different parts of the Amazon; they found that the intensity trends of the two fluorescent substances in the soil HA are the same.

In this study, we conducted a 12-month field trial, after the same amount of corn straw was returned to the soil in different returning modes. The differences in characteristics of the soil HA were studied by fluorescence spectroscopy combined with infrared (IR) spectroscopy. Additionally, the degree of aromatization and humification of soil HA was evaluated.

## 2. Materials and Methods

### 2.1. Soil and Corn Straw

The field trial was conducted at the Corn Experimental Station of Jilin Agricultural University. The climate type was the temperate continental monsoon climate with an annual average temperature of 5.6°C, frost-free period of 135–155 d, precipitation of 500–600 mm, and sunshine duration of 2600 h; the annual accumulated temperature is 3200°C. The tested soil had been subjected to continuous cropping field which is equivalent to the Argiudolls of the US system classification. The basic properties of the soil are listed in Table 1. The test crop residue was a mature corn straw, about 3–5 cm after pulverization; the properties of the straw were as follows: organic carbon 338.6 g/kg, total nitrogen 6.87 g/kg, and C/N of 49.3 : 1.

### 2.2. Corn Straw Returning and Humic Acids

The test was started on April 15, 2016. The amount of straw returned to the field was 8974.3 kg/hm^2^, and urea was applied at 400 kg/hm^2^ (the C/N ratio was adjusted to 20 : 1). Five treatments were set up in the experiment: CK (control): no straw was applied; St: corn straw was evenly placed in the topsoil (5–20 cm), and the surface was covered with topsoil; Ss: corn straw was evenly placed in the subsoil (20–40 cm), and the upper soil was covered successively; Smt: corn straw was uniformly mixed with topsoil, and then, the topsoil was covered; Sms: corn straw was evenly mixed with subsoil, and then, the upper soil was covered successively. Three points were randomly selected for each treatment to collect soil samples (mixing the soil from the 3 points) on April 15, 2017, and the soil samples were air-dried to remove organic residues prior.

Soil HAs were extracted and purified following the IHSS method [32]: A portion of the air-dried soil sample (100 g), which had passed through a 2 mm sieve was weighed into a 2.5 L glass bottle; 1 mol/L HCl was added, followed by dilution at a ratio of 1 : 1 with water; and the resultant solution was kept for 1 h. The final sample was diluted to a ratio of 1 : 10 with 0.1 mol/L HCl. After neutralizing with 1 mol/L NaOH to achieve pH = 7, the solution was centrifuged in a large centrifuge tube and kept for 1 h; the precipitate was washed into a glass bottle with 0.1 mol/L NaOH and adjusted to a final sample water ratio of 1 : 10. Nitrogen was immediately (1 min) added to the sample, which was covered tightly. The next day, the mixture was centrifuged, and subsequently, the supernatant was transferred into another bottle, and the extraction was carried out for three times. The pH of the supernatant was adjusted to 1.0 with 6 mol/L HCl, kept for 12–16 h, and centrifuged, after which the supernatant was discarded. The sample was dissolved in 30 mL of HCl (0.1 mol/L) + HF (0.3 mol/L) in a centrifuge tube, shaken at room temperature overnight, and centrifuged, after which the supernatant was discarded. The pickling step was repeated 3 times. After centrifugation, Cl^−^ was removed by electrodialysis, and finally, the purified HA sample was obtained by rotary evaporation and lyophilization to remove water.

### 2.3. IR Spectroscopy Analysis of Humic Acids

The Fourier transform infrared spectrometer (Brucker TENSOR 27, German) was used to analyze the HAs, and the wave number was ranged from 4000 to 500 cm^−1^, following the KBr tablet method. The relative intensity of each IR vibration was calculated by integrating the peak area using the OMNIC software.

### 2.4. Fluorescence Spectroscopy Analysis of Humic Acids

The fluorescence spectra were recorded by using a FL 6500 fluorescence spectrometer (Perkin Elmer, USA) equipped with a 550 V voltage photomultiplier tube (PMT) on aqueous solutions (dissolved in Milli-Q water) of 35 mg/L HA after overnight equilibration at a temperature 25°C and adjustment to pH 8.0 with 0.05 mol/L NaHCO_3_. The emission spectra were recorded over the range of 380–650 nm at a constant excitation wavelength of 360 nm, and the excitation spectra were recorded over the range of 280–500 nm at a fixed emission wavelength of 520 nm [22, 25]. The synchronous-scan fluorescence spectra were obtained at the condition of Δ*λ* = 55 nm and a scanning speed of 200 nm/min. The EEM spectra of HAs were collected with subsequent scanning emission spectra from 350 to 700 nm by varying the excitation wavelength from 270 to 550 nm at 10 nm increments. Both of the excitation and emission band-pass widths were 10 nm, and the scan speed was 2400 nm/min. All the spectra were obtained by subtracting the spectra of Milli-Q water as the blank and finally conducting the analysis using the Origin75 software.

## 3. Results and Discussion

### 3.1. IR Characteristics of HAs

**Figure 1:**
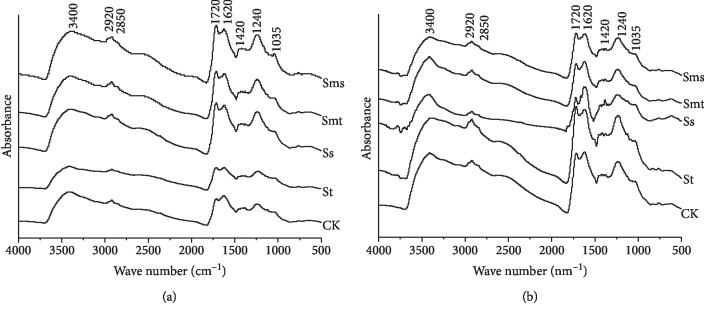
Infrared spectra of humic acids: (a) topsoil (5–20 cm); (b) subsoil (20–40 cm). No straw was applied (CK), straw returning to topsoil (St), straw returning to subsoil (Ss), straw mixing with topsoil (Smt), and straw mixing with subsoil (Sms).

The IR spectra of soil HAs of different treatments are shown in Figure 1, and the relative intensities of each vibration are listed in Table 2. IR bands were assigned according to Stuart et al. [33] and Günzler and Gremlich [34] as follows: bands around 2920 cm^−1^ and 2850 cm^−1^ were attributed to the symmetric and asymmetric stretching vibrations of aliphatic C–H bonds in CH_3_ and CH_2_ groups; the band at 1720 cm^−1^ was attributed to the C=O stretching vibration, mainly caused by COOH groups, and also to other carbonyl groups, such as ketones and aldehydes. The band at 1620 cm^−1^ is attributed to the C=C vibrations of aromatic structures. The band at 1035 cm^−1^ corresponds to the C–O stretching vibration of the carbohydrate or polysaccharide structure and the Si–O stretching vibration of the inorganic substance. The peak area integral ratio of the peaks at 2920 cm^−1^ and 1720 cm^−1^ can be used to characterize the oxidation degree of HA; the ratio of 2920/1620 can indicate the aliphaticity and aromaticity of HA.

For topsoil HAs, the relative vibration intensities of 1035 cm^−1^ and 1720 cm^−1^ show the same trend: St > Sms > Ss > Smt > CK. This indicates that the structure of the topsoil HA after straw returning contains more simple structures, such as carbohydrates, and the number of carboxyl groups is also increased. The two indicators of Smt and Ss HA are closer to CK. At the ratio of 2920/1720, CK > Smt > St > Ss > Sms; this trend shows that Sms can improve the oxidation degree of the topsoil HA. At the ratio of 2920/1620, St > Sms > Smt > Ss > CK; straw returning resulted in a decrease in the degree of aromatization of the topsoil HA, which was associated with the decomposition of the straw applied to the topsoil to produce simple compounds. Among the 4 straw returning treatments, Ss is the most beneficial for improving the aromatization degree of the topsoil HA and the aromatization degree of St HA in the topsoil was the lowest. Considering that the straw decomposed quickly when the corn straw was placed in the topsoil, it can decompose more substances with simple structures.

For subsoil HAs, the changes in the relative absorption intensity of the HAs treated by straw returning were noticeable, although the trend was different. At the vibrations of 1720 cm^−1^, St > Sms > CK > Smt > Ss; a significant reduction in the number of carboxyl groups in Ss HA and Smt HA was observed; at the vibrations of 1035 cm^−1^, St > CK > Sms > Ss > Smt. The amounts of carbohydrates in Ss HA and Smt HA are lower than that of CK, which may indicate that the two modes of straw returning complicate the structure of the subsoil HA. At the ratio of 2920/1720, Ss > Smt > St > CK > Sms; this result indicates that Ss HA has the lowest oxidation degree. At the ratio of 2920/1620, St > CK > Smt = Sms > Ss; this indicates that Ss HA has the highest degree of aromatization. It was speculated that the decrease in the carboxyl content may be due to the fact that Ss and Smt treatment can promote the bonding of carboxyl and straw decomposing products in soil HA to form more complex materials.

### 3.2. Fluorescence Spectra Analysis

#### 3.2.1. Emission Fluorescence Spectra

**Figure 2:**
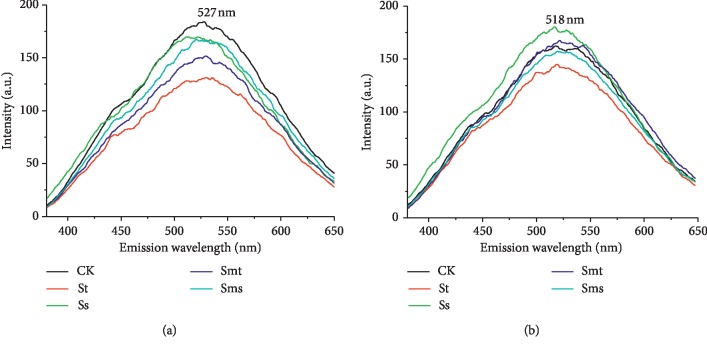
Emission fluorescence spectra of humic acids: (a) topsoil (5–20 cm); (b) subsoil (20–40 cm). No straw was applied (CK), straw returning to topsoil (St), straw returning to subsoil (Ss), straw mixing with topsoil (Smt), and straw mixing with subsoil (Sms).

The monodimensional fluorescence spectra in the emission mode of the HAs are shown in Figure 2; the peak position and intensities (in arbitrary units (a.u.)) are listed in Table 3. Our results showed a similar emission spectrum for each sample, and the emission spectra featured a typical unique broad band with a flat maximum whose wavelength was positioned at 527 nm (topsoil HA) and 518 nm (subsoil HA). Milori et al. [24] found that a high fluorescence intensity is correlated with high humification. For topsoil HAs, the emission peak intensities of straw returning HAs were all weaker than those of CK HA, i.e., CK > Ss > Sms > Smt > St. This indicated that straw returning resulted in a decrease in the intensity of the fluorescent peak and a decrease in the degree of humification in the topsoil. In the subsoil, the intensities of the emission peak of the HAs were in the order of Ss > Smt > CK > Sms > St; this indicates that Ss and Smt will increase the number of fluorescent substances and increase the degree of humification in the subsoil HA.

#### 3.2.2. Excitation Fluorescence Spectra

**Figure 3:**
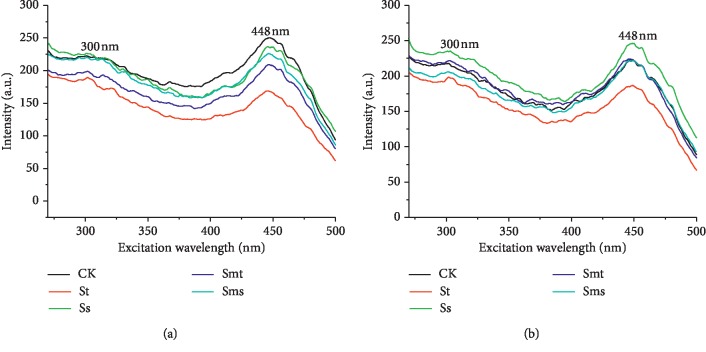
Excitation fluorescence spectra of humic acids: (a) topsoil (5–20 cm); (b) subsoil (20–40 cm). No straw was applied (CK), straw returning to topsoil (St), straw returning to subsoil (Ss), straw mixing with topsoil (Smt), and straw mixing with subsoil (Sms).

The monodimensional fluorescence spectra in the excitation mode of the HAs are shown in Figure 3, and the peak position and intensities are listed in Table 3. The excitation spectra had one main peak at 448 nm and a less intense peak at 330 nm. This indicates that the soil HAs contain at least two different structures of fluorescent substances. Some researchers have obtained similar results when performing fluorescence analysis on soil HA [25]. Senesi et al. [23] believed that the blue shift (moving toward short wavelengths) of the fluorescence peak indicates a reduction in the degree of humification, whereas the red shift of the fluorescence peak (moving toward long wavelengths) indicates an increase in the humification degree. Milori et al. [24] found a peak at 465 nm in the excitation spectrum of soil HA, which is similar to the peak at 448 nm in this study, and the peak shapes are similar. This peak is probably associated with more humified structures because it locates at long wavelength. The peak of 300 nm is indicative of a fluorescent substance with a humification degree lower than that indicated by the 448 nm peak.

In the excitation spectra of topsoil HAs, the order of fluorescence intensity at 448 nm is CK > Ss > Sms > Smt > St. In subsoil HA, the order of fluorescence intensity at 448 nm is Ss > Smt > CK > Sms > St. This result is similar with the intensity trend of emission spectra. It is shown that the effects of four straw returning modes on the excitation and emission spectra of soil HA are the same.

#### 3.2.3. Synchronous-Scan Fluorescence Spectra

**Figure 4:**
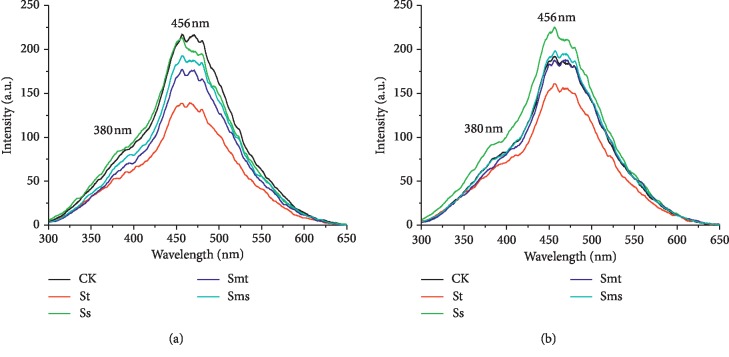
Synchronous fluorescence spectra of humic acids: (a) topsoil (5–20 cm); (b) subsoil (20–40 cm). No straw was applied (CK), straw returning to topsoil (St), straw returning to subsoil (Ss), straw mixing with topsoil (Smt), and straw mixing with subsoil (Sms).

The monodimensional fluorescence spectra in the synchronous-scan modes of the HAs are shown in Figure 4, and the peak position and intensities are listed in Table 3. The basic idea of Zsolnay et al. [35] is that as fluorescing molecules become more condensed, their emission spectra will tend to red shift. Predicting the degree of humification according to the wavelength shift is easy to make the conclusion inaccurate. Zsolnay et al. [35] proposed that *A*_4_/*A*_1_ (the area in the upper quarter of emission spectra divided by the area in the lower quarter) can be used to evaluate the humification. Because this method is trouble to calculate, with the method proposed by Kalbitz et al. [36], the synchronous spectra were measured while keeping a constant wavelength difference, Δ*λ* = *λ*_em_ − *λ*_ex_ = 55 nm. According to Kalbitz et al. [36], the synchronous fluorescence spectra of the HA present a peak at around 470 nm and a shoulder at around 360 nm. These profiles change depending on the humification degree, and this change can be measured through fluorescence peak ratios. The shift in the maximum fluorescence intensity from shorter to longer wavelengths is associated with an increasing number of highly substituted aromatic nuclei and/or with a conjugated unsaturated system capable of a high degree of resonance. Thus, the ratio of fluorescence intensity at 470 nm and 360 nm (I_470_/I_360_) was used to measure the degree of HA polycondensation or humification. Santos et al. [31] used the condition of Δ*λ* = 55 nm in the study of synchronous-scan fluorescence of soil HA and used the intensity ratio at 460 nm and 378 nm (I_460_/I_378_) to evaluate the degree of humification of HA. Milori et al. [24] adapted the intensity ratio of 465 nm and 399 nm (I_465_/I_399_) to examine the degree of humification of soil HA also at Δ*λ* = 55 nm, and the results were consistent with the consequences obtained by electron spin resonance spectroscopy. In this study, there is one main peak at 456 nm and a shoulder at 380 nm in the synchronous fluorescence spectrum. Here, the ratio of I_456_/I_380_ is used to evaluate the humification degree and is listed in Table 3.

For topsoil HAs, the order of I_456_/I_380_ is CK > Ss > Sms > Smt > St. This is consistent with the order of excitation and emission intensity, and it indicates that the humification degree of the topsoil HA is reduced in all four modes of straw returning. For subsoil HAs, the order of I_456_/I_380_ is Ss > Smt > CK > Sms > St, indicating that Ss and Smt treatment can increase the degree of humification of the subsoil HA. The treatment of Ss in the four modes is most beneficial to the humification of the subsoil HA.

#### 3.2.4. Total Luminescence Spectra of Humic Acids

**Figure 5:**
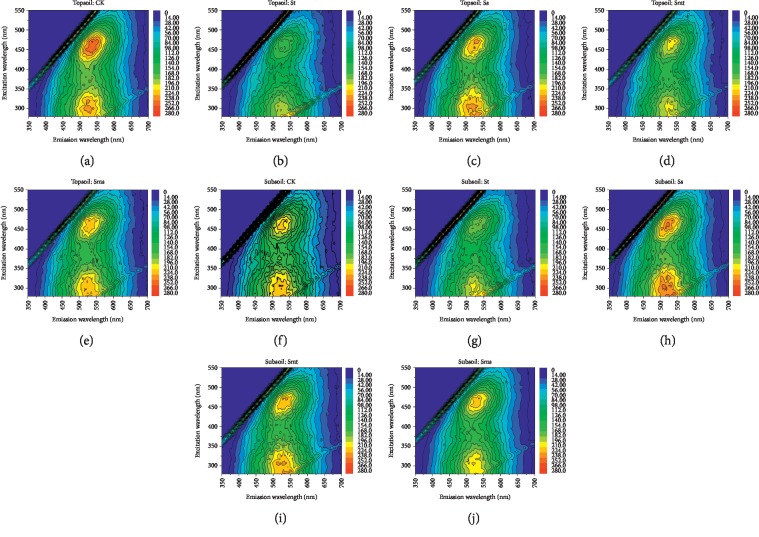
Total luminescence spectra of humic acids: topsoil (5–20 cm); subsoil (20–40 cm). No straw was applied (CK), straw returning to topsoil (St), straw returning to subsoil (Ss), straw mixing with topsoil (Smt), and straw mixing with subsoil (Sms). (a) Topsoil: CK. (b) Topsoil: St. (c) Topsoil: Ss. (d) Topsoil: Smt. (e) Topsoil: Sms. (f) Subsoil: CK. (g) Subsoil: St. (h) Subsoil: Ss. (i) Subsoil: Smt. (j) Subsoil: Smt.

The TL spectra in the form of EEM (or contour maps) of the HAs are shown in Figure 5, and the excitation/emission wavelength pairs (EEWP) of the main peak maximum and fluorescence intensities are listed in Table 4. The principal peak (*λ*_ex_/*λ*_em_ = 430–510 nm/470–580 nm) appeared in the HA spectra of all treatments (including CK), which had long excitation and emission wavelengths. According to Matthews et al. [37], this is a characteristic peak of HA derived from lignin (denominated peak L) which has an extended, highly polymerized linear aromatic ring structure, and other highly humidified macromolecules with electron-donating groups such as hydroxyl, methoxy, or amino. Coble [20] proposed that the peak in the HA spectra located at *λ*_ex_/*λ*_em_ = 290–345 nm/450–560 nm in our study should be represented by the symbol C, while Parlanti et al. [38] marked this peak with *β*. Some scholars have regarded this peak as being indicative of a phenol-like or naphthol-like structure [39]. Here, this peak was also indicated by the symbol, C.

In the topsoil, in Figure 4, the peak L intensities of the HAs treated with straw returning were lower than that of CK, and the trend was consistent with the change of the emission spectra: CK > Ss > Sms > Smt > St. For peak C, the intensity order is the same with peak L. This phenomenon indicates that straw returning can decrease the number of lignin-like structures (with electron-donating groups) and the number of phenol-like or naphthol-like structures in the topsoil HA. This result shows that straw returning simplifies the structure of the topsoil HA, which is consistent with that obtained from the monodimensional fluorescence spectra analysis results.

In the subsoil, for peak L, the intensity is in the order of Ss > Smt > Sms > CK > St, and for peak C, the intensity order is Ss > Smt > CK > Sms > St. The intensities of peak L and peak C in Ss HA are the highest; one reason is due to the increased number of lignin-like structures (with electron-donating groups), and the other is attributed to the inclusion of more phenolic structures. Smt HA has the same result but the intensity is slightly reduced. It is concluded that straw returning to the subsoil majorly simplifies the phenol-like or naphthol-like structure of the subsoil HA and complicates the lignin-like structure, which is consistent with the relatively high degree of aromatization of St HA in the IR spectra.

## 4. Conclusions

Fluorescence spectroscopy and IR spectroscopy were adapted to analyze the structure of soil HA after straw returning at four different modes. The IR spectra showed the number of carboxylic groups, aliphatic character, aromatization degree, and oxidation degree of HA. According to the monodimensional fluorescence spectra, the peak wavelengths and intensities of HA can indicate the complication and amount of fluorescent substance. The EEM spectra showed the information of the lignin-like structure (with electron-donating groups) and naphthol-like structure in the soil HA. In addition, the ratio of I_456_/I_380_ calculated from the synchronous-scan fluorescence spectra can be used to evaluate the humification of soil HA. It is concluded that Ss is the most beneficial mode for improving the humification and aromatization degree of subsoil HA, increasing the number of carboxylic groups in topsoil HA and the naphthol-like structure and lignin-like structure in the subsoil HA. It is recommended that the mode of straw returning to subsoil is employed when farming because it will help increase the carbon sequestration of the subsoil and the humification of the HA in the subsoil, which is beneficial for crop growth.

In conclusion, fluorescence spectroscopy is demonstrated to be a useful technique for evaluating the degree of humification and the fluorescent structure of soil HA. Compared with other methods, fluorescence spectroscopy and IR spectroscopy are relatively simple techniques that use low-cost tools for studying soil HA.

## Figures and Tables

**Table 1:** Basic properties of the soil tested in the experiment.

Depth (cm)	Organic carbon (g/kg)	Available N (mg/kg)	Available P (mg/kg)	Available K (mg/kg)	pH
5–20	19.44	78.97	24.08	97.67	6.56
20–40	18.63	69.19	21.31	85.56	6.48

**Table 2:** Characteristic peaks of infrared spectra of soil humic acids.

Treatment	Depth (cm)	Relative intensity					Ratio	
		2920 (cm^−1^)	2850 (cm^−1^)	1720 (cm^−1^)	1620 (cm^−1^)	1035 (cm^−1^)	2920/1720	2920/1620
CK	5–20	0.918	0.447	7.550	12.510	1.129	0.181	0.109
St		0.917	0.481	14.608	6.730	2.347	0.096	0.208
Ss		0.835	0.450	14.111	9.510	1.908	0.091	0.135
Smt		0.967	0.341	12.722	9.895	1.531	0.103	0.132
Sms		0.859	0.367	14.364	9.041	2.251	0.085	0.136

CK	20–40	0.772	0.384	12.742	9.176	1.846	0.091	0.126
St		0.970	0.540	14.985	10.360	2.144	0.101	0.146
Ss		1.061	0.509	4.391	16.014	1.633	0.358	0.098
Smt		0.838	0.432	9.524	11.166	1.165	0.133	0.114
Sms		0.804	0.367	14.127	10.269	1.710	0.083	0.114

Note: 2920/1720 means (2920 + 2850)/1720; 2920/1620 means (2920 + 2850)/1620. No straw was applied (CK), straw returning to topsoil (St), straw returning to subsoil (Ss), straw mixing with topsoil (Smt), and straw mixing with subsoil (Sms).

**Table 3:** Typical properties of monodimensional fluorescence spectra of soil humic acids at different straw returning modes.

Treatment	Soil depth (cm)	Emission	Excitation		Synchronous-scan		
		527/518 nm intensity (a.u.)	300 nm intensity (a.u.)	448 nm intensity (a.u.)	380 nm intensity (a.u.)	456 nm intensity (a.u.)	I_456_/I_380_
CK	5–20	183.5	222.3	249.3	74.8	215.8	2.89
St		130.2	188.2	167.5	53.5	138.2	2.58
Ss		168.3	226.3	236.6	78.2	212.9	2.72
Smt		150.7	198.5	208.8	63.5	166.2	2.62
Sms		165.8	219.2	225.4	68.9	182.1	2.64

CK	20–40	161.5	216.5	222.4	73.6	192.2	2.61
St		143.4	197.5	185.7	60.7	155.5	2.56
Ss		179.6	235.6	245.9	85.0	230.4	2.71
Smt		164.9	221.7	223.9	68.7	183.2	2.67
Sms		155.1	205.5	221.7	73.5	190.7	2.59

Note: no straw was applied (CK), straw returning to topsoil (St), straw returning to subsoil (Ss), straw mixing with topsoil (Smt), and straw mixing with subsoil (Sms).

**Table 4:** Positions and intensities of peaks in total luminescence fluorescence spectra of soil humic acids.

Treatment	Depth (cm)	Peak L		Peak C	
		EEWP (nm)	Intensity (a.u.)	EEWP (nm)	Intensity (a.u.)
CK	5–20	472/536	254.0	307/521	231.1
St		473/524	171.7	307/518	195.1
Ss		473/532	235.6	307/522	230.0
Smt		473/528	216.6	307/521	202.8
Sms		472/531	223.7	307/520	225.3

CK	20–40	473/531	220.4	307/528	225.9
St		473/533	188.9	307/523	208.7
Ss		473/536	243.6	307/530	243.0
Smt		472/538	231.2	307/533	232.0
Sms		473/536	226.2	307/520	213.9

Note: no straw was applied (CK), straw returning to topsoil (St), straw returning to subsoil (Ss), straw mixing with topsoil (Smt), and straw mixing with subsoil (Sms).

## Data Availability

All data generated or analyzed during this study are included in this article.
